# Danger in the Water Trough

**Published:** 1885-03

**Authors:** 


### DANGER IN THE WATER TROUGH.

The *British Medical Journal* suggests a danger to horses at public drinking troughs. It believes that glanders are spread among horses in this way, and recommends a stand pipe and bucket as the safest and best arrangement for watering animals in cities. It is more comfortable for the horse, who has not to strain his neck against the collar to reach the water, the water is fresher and more palatable, and there is far less danger of its being contaminated with dust, dirt, and the germs of disease.

